# Transiente epileptische Amnesie – ein seltenes Phänomen bei Temporallappenepilepsien

**DOI:** 10.1007/s00115-022-01364-5

**Published:** 2022-08-03

**Authors:** Jan Pukropski, Randi von Wrede, Christoph Helmstaedter, Rainer Surges

**Affiliations:** Klinik und Poliklinik für Epileptologie, Universitätsklinikum Bonn, Venusberg-Campus 1, 53127 Bonn, Deutschland

**Keywords:** Schläfenlappenepilepsie, Differenzialdiagnose, Transiente Gedächtnisstörungen, Transiente globale Amnesie, Erwachsene, Temporal lobe epilepsies, Differential diagnosis, Transient memory disturbances, Transient global amnesia, Adults

## Abstract

**Hintergrund:**

Die transiente epileptische Amnesie (TEA) ist ein seltenes Phänomen bei Temporallappenepilepsien, das häufig nicht erkannt oder als transiente globale Amnesie (TGA) fehldiagnostiziert wird. Als Ursache werden iktale und postiktale Störungen der Gedächtnisbildung postuliert, was auch durch das Ansprechen auf Antiepileptika gestützt wird. Angesichts der zunehmenden Zahl neu auftretender Epilepsien im höheren Lebensalter ist auch mit einer Zunahme der TEA zu rechnen.

**Ziel der Arbeit:**

Analyse typischer Merkmale der TEA in einer monozentrischen Fallserie.

**Material und Methoden:**

Mittels interner Datenbankanalyse wurden unter 7899 Patient*innen über einen Zeitraum von 8 Jahren 10 Patient*innen mit TEA identifiziert. Klinische Merkmale sowie Befunde der Zusatzdiagnostik wurden retrospektiv untersucht. Die Daten sind als Mittelwert ± SD angegeben.

**Ergebnisse:**

Bei allen 10 Patient*innen wurde die Diagnose einer Temporallappenepilepsie gestellt. Das Lebensalter bei Erstmanifestation der TEA lag bei 59,1 ± 6,7 Jahren, die Diagnose wurde mit einer Latenz von 21,9 ± 26,3 Monaten gestellt. Eine TEA-Episode dauerte 56 ± 37 min an und trat pro Jahr 16 ± 9,9-mal auf; 6 von 10 Patient*innen berichteten über häufiges Auftreten direkt nach dem Erwachen. Bei 9 von 10 Patient*innen wurde über weitere Anfallstypen bzw. weitere semiologische Elemente während der TEA berichtet. Hinweise auf neuropsychologische Störungen temporaler Funktionen ergaben sich bei 8 von 10 Patient*innen, Hinweise auf eine depressive Störung bei 6 von 10 Patient*innen. Im Schlaf aktivierte epilepsietypische Aktivität wurde bei 4 Patient*innen temporal links sowie bei 2 Patient*innen temporal beidseits nachgewiesen. Bei 3 Patient*innen wurden mittels Kernspintomographie typische Auffälligkeiten im Bereich der temporomesialen Strukturen (bei 2 links, bei 1 rechts) nachgewiesen. Eine antiepileptische Therapie verbesserte die Anfallskontrolle bei 7 von 10 Patient*innen (Anfallsfreiheit bei 6 Patienten), bei 3 Patienten ist die therapeutische Wirkung unbekannt.

**Diskussion:**

TEA sind selten, treten im höheren Erwachsenenalter auf und werden erst nach etwa 2 Jahren korrekt als epileptisches Phänomen diagnostiziert. Die gründliche Erfassung von Begleitsymptomen, die Umstände und das rezidivierende Auftreten sowie Hinweise auf eine Temporallappenepilepsie in den apparativen Zusatzuntersuchungen ermöglichen die Differenzierung zur TGA.

## Abstract (en)

**Background:**

Transient epileptic amnesia (TEA) is a rare phenomenon in temporal lobe epilepsy that is often unrecognized or misdiagnosed as transient global amnesia (TGA). It is postulated that TEA is due to both ictal and postictal disturbances. Response to antiseizure medication underlines its epileptic nature. In view of the increasing incidence of new-onset epilepsies in old age, an increase in TEA can be expected in the future.

**Objective:**

Analysis of TEA features in a monocentric case series.

**Material and methods:**

A search in our electronic patient data base yielded 10 patients with TEA out of 7899 patients over a period of 8 years. Clinical and paraclinical features as well as findings of additional examinations were retrospectively collected. Data are given as mean ± SD.

**Results:**

All 10 patients were diagnosed with temporal lobe epilepsy. The mean age at manifestation of TEA was 59.1 ± 6.7 years, the diagnosis was made with a delay of 21.9 ± 26.3 months. The TEA lasted on average 56 ± 37 min, and 16 ± 9.9 TEA episodes per year were reported by the patients; out of the 10 patients 6 reported that TEA usually occurred upon awakening. In 9 of 10 patients, there was evidence of typical seizure symptoms or other semiological elements during TEA. Interictal neuropsychological disturbances of temporal functions were seen in 8 of 10 patients and evidence of depressive disorder in 6 of 10 patients. Video EEG recordings revealed epileptiform activity during sleep in 4 patients over the left and in 2 patients over both temporal regions. In 3 patients, magnetic resonance imaging displayed typical alterations of the temporomesial structures (in 2 patients on the left and in 1 the right side). Antiseizure medication improved seizure control in 7 of 10 patients (seizure freedom in 6 patients), 3 patients were lost to follow-up.

**Discussion:**

TEA is rare, occurs in older adults and is correctly diagnosed after about 2 years. Thorough assessment of additional symptoms and circumstances, the recurrent occurrence as well as typical EEG and imaging findings of temporal lobe epilepsy enables the distinction between TEA and TGA.

## Einleitung

Die transiente epileptische Amnesie (TEA) ist ein seltenes Phänomen bei Patient*innen mit Temporallappenepilepsie, das oft nicht als solches erkannt oder als transiente globale Amnesie (TGA) verkannt wird. Häufig wird die TEA nach dem Erwachen berichtet und dauert typischerweise über 15–60 min an [1, 2]. Die TEA ist durch eine gemischte anterograde und retrograde Gedächtnisstörung charakterisiert. Nicht selten werden Betroffene auch durch Stellen repetitiver Fragen auffällig. Patient*innen mit TEA sind meist im mittleren Lebensalter und haben darüber hinaus oft auch permanente Gedächtnisstörungen (v. a. akzeleriertes Vergessen, autobiografische und räumlich-topografische Gedächtnislücken), zeigen eine emotionale Labilität und sind depressiv verstimmt. Im Gegensatz zur TGA treten TEA regelhaft rezidivierend auf (durchschnittlich einmal pro Monat) und respondieren auf Antiepileptika [1, 2]. Klinisch lassen sich bei TEA-Patient*innen häufig auch andere Merkmale von Temporallappenanfällen eruieren und in weiterführenden Untersuchungen können typische Befunde erhoben werden (in etwa einem Drittel interiktale epileptiforme Aktivität über den Temporallappen, bei wenigen auch kernspintomographische Läsionen im Temporallappen; [2]). In der vorliegenden Arbeit werden klinische und paraklinische Merkmale von 10 Patient*innen mit TEA beschrieben und im Kontext des aktuellen Wissenstands diskutiert. Darauf basierend wird ein klinisches Vorgehen zur korrekten Diagnosestellung von TEA abgeleitet.

## Patienten und Methoden

### Ein- und Ausschlusskriterien

In die Fallserie wurden alle Patienten eingeschlossen, die zwischen den Jahren 2014 und 2021 an der Klinik und Poliklinik für Epileptologie stationär behandelt wurden und die in der elektronischen Patientendatenbank durch die Schlagwortsuche „epileptische Amnesie“ und „transiente epileptische Amnesie“ retrospektiv identifiziert wurden. Neben dem wiederholten Auftreten einer paroxysmalen amnestischen Störung wurde auch nach typischen Befunden bei Epilepsien gesucht, nämlich dem EEG-Nachweis epilepsietypischer Potenziale, dem Nachweis epileptogener Läsionen in der kranialen Kernspintomografie, dem Bericht über weitere semiologische Elemente von fokalen Anfällen aus dem Temporallappen (z. B. aufsteigende Übelkeit oder Unwohlsein, olfaktorische oder gustatorische Auren, Angstauren, Piloarrektion, orale oder manuelle Automatismen) oder indirekten Hinweisen auf stattgehabte fokal zu bilateral tonisch-klonische Anfälle.

Die Einschlusskriterien orientieren sich an den im Jahr 1998 von Zeman beschriebenen typischen Merkmalen einer TEA [17] (s. Infobox 1).

#### Infobox 1 Diagnosekriterien der TEA nach Zeman

- Wiederholte Episoden mit Amnesie
- Weitere kognitive Funktionen während typischer Episoden unbeeinträchtigt
- Weitere Hinweise auf Epilepsie (epilepsietypische Potenziale, andere Elemente epileptischer Anfälle wie Auren oder Automatismen)
- Ansprechen auf eine antiepileptische Therapie

Patient*innen ohne Hinweise auf sich wiederholende amnestische Episoden oder ohne Hinweise auf eine Epilepsie wurden nicht in die Fallserie eingeschlossen. Auch Patient*innen mit einer Amnesie mit begleitender Bewusstseinsstörung, Vigilanzminderung (wie z. B. häufig nach tonisch-klonischen Anfällen) oder Störung weiterer kognitiver Funktionen (z. B. Sprachstörung) wurden ausgeschlossen.

Deskriptive statistische Daten werden als Mittelwert ± Standardabweichung (SD) angegeben.

## Ergebnisse

### Klinische Merkmale der Patient*innen mit TEA

Unter 7899 Patient*innen wurden 10 Patient*innen mit TEA identifiziert. Neun von 10 Patient*innen waren männlich. Das Alter bei Erstmanifestation der TEA variierte zwischen 46 und 69 Jahren (59,1 ± 6,7 Jahre), 9 von 10 Patient*innen waren bei Erstmanifestation älter als 50 Jahre. Die zeitliche Latenz zwischen Erstmanifestation und Diagnosestellung betrug 21,9 ± 26,3 Monate. Interessanterweise hatten 5 von 10 Patient*innen einen technischen Beruf erlernt (Tab. 1).

**Table 1:** 

Fall	m/w	Alter bei Erstmanifestation (Jahre)	Alter bei Diagnose (Jahre)	Dauer bis Diagnosestellung in Monaten (Jahr)	Beruf	Gedächtnis	Depression
1	m	68	69	10 (2014)	Techniker	+	+
2	m	69	72	25 (2017)	Techniker	+	–
3	m	56	57	23 (2017)	Techniker	+	+
4	m	54	55	18 (2020)	Techniker	++	+
5	m	58	59	10 (2020)	Angestellter	++	++
6	m	46	47	13 (2020)	Angestellter	–	(+)
7	w	66	67	16 (2020)	Krankenpflegerin	–	–
8	m	58	58	1 (2021)	Metzger	–	–
9	m	60	68	98 (2021)	Techniker	+	+
10	m	56	57	5 (2021)	Angestellter	+	–

*m/w* männlich/weiblich, + leicht, ++ mittelgradig, (+) belastet, – keine Störung

Sieben von 10 Patient*innen berichteten über subjektive Gedächtnisstörungen, welche bei 2 Patienten zu Einschränkungen und Problemen im Alltag führten. Eine subjektive – teilweise mit einem Antidepressivum behandelte – depressive Störung wurde von 5 Patienten berichtet und war bei 4 Patienten leichtgradig und bei 1 Patienten mittelgradig ausgeprägt. Ein weiterer Patient fühlte sich durch die regelmäßig auftretenden Anfälle (24/Jahr) stark belastet (Tab. 1).

Auswärtig wurde bei 3 Patienten bereits eine Temporallappenepilepsie vermutet, bei 1 Patienten wurden Vigilanzschwankungen diagnostiziert. Bei 3 Patient*innen wurde eine Amnesie unklarer Ursache als Diagnose formuliert, und bei jeweils 1 Patienten eine TGA, eine Verwirrtheitsepisode und dissoziative Anfälle.

### Exemplarische Fallbeschreibung: Fall 6

**Figure 1:**
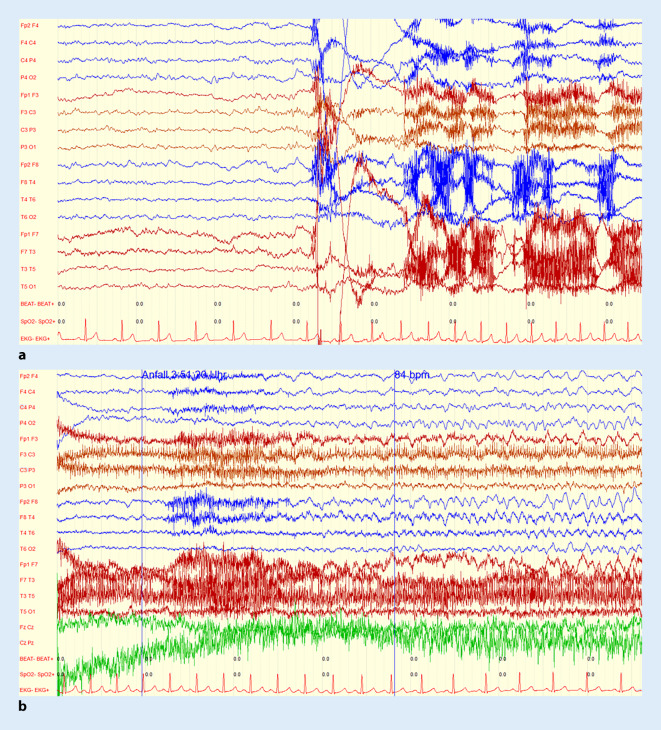

47-jähriger Patient, kaufmännischer Angestellter. Bei Vorstellung berichtete der Patient, dass er vor einem Jahr erstmalig eine amnestische Episode über ca. 30 min nach dem morgendlichen Erwachen bemerkt habe. Er habe den Tag und die Tagesplanung nicht erinnern können, auch seien ihm die Ereignisse des Vortags sowie seine Handy-PIN nicht erinnerlich gewesen. Fremdanamnestisch habe sich der Patient unauffällig verhalten, im Verlauf seien jedoch auch Episoden mit Areagibilität und starrem Blick beobachtet worden. Danach seien in Abständen von ca. 3 Wochen immer morgens nach dem Aufwachen ähnliche Episoden über bis zu 60 min aufgetreten. Eine auswärtige stationäre Diagnostik mittels CCT-, cMRT-, Ruhe-Wach-EEG-Untersuchung (auch nach Schlafentzug) sowie einer Liquordiagnostik waren nicht wegweisend gewesen. Während einer Video-EEG-Ableitung in unserer Klinik konnten am frühen Morgen nach dem Erwachen 2 amnestische Episoden mit einem Herzfrequenzanstieg aufgezeichnet werden, wobei sich bei einer Episode ein Anfallsmuster über 40 s beginnend mit einer rhythmischen Thetaaktivität frontotemporal rechts zeigte (Abb. 1). Der erste Anfall wurde von dem Patienten selbst nicht bemerkt und auch nicht erinnert. Beim zweiten Anfall ohne Nachweis eines Anfallsmusters schrieb der Patient seiner Ehefrau eine Kurznachricht mit der Frage „Wo er hier [sei] und was er hier [mache]?“. Erst fünf Minuten später bemerkte er, dass er einen Anfall gehabt hatte. Interiktal zeigten sich im Schlaf epilepsietypische Potenziale temporal rechts. In einer cMRT-Untersuchung war keine epileptogene Läsion nachweisbar. In der neuropsychologischen Untersuchung ergaben sich Hinweise auf eine Funktionsstörung temporal rechts. Das Depressionsinventar verwies auf eine depressiv gefärbte Stimmungslage. Die antineuronalen Antikörper waren negativ. Es wurde eine Temporallappenepilepsie rechts unklarer Ursache diagnostiziert und eine antiepileptische Therapie mit Lamotrigin eingeleitet. In einer Follow-up-Untersuchung nach 6 Monaten berichtete der Patient über durchgehende Anfallsfreiheit.

### Anfallsmerkmale

Die Frequenz der berichteten Anfälle variierte zwischen 4 und 36 Anfällen pro Jahr (16 ± 9,9). Die Dauer eines Anfalls lag meist zwischen 15 und 60 min (56 ± 37 min); bei Fall 10 wurde eine TEA mit einer Dauer von 150 min berichtet. Bei 5 Patient*innen kam es zum Auftreten von Anfällen am Morgen. Insgesamt berichteten 6 Patient*innen über ein Auftreten der TEA direkt nach dem Erwachen. Von einem nächtlichen Auftreten aus dem Schlaf heraus oder nach nächtlichem Erwachen wurde von keiner Patient*in berichtet. Bei 9 Patient*innen ergab die Anamnese Hinweise auf weitere Anfallstypen bzw. auf weitere semiologische Elemente während des Anfalls (Tab. 2). Drei Patient*innen erhielten bereits auswärtig die Diagnose einer Epilepsie.

**Table 2:** 

Fall	AF/Jahr	TEA-Dauer (min)	Tageszeitlicher Schwerpunkt	Nach Erwachen	Weitere Semiologie oder andere Anfallstypen
1	12	20	Tag	–	1 × bilateral tonisch-klonischer Anfall
2	36	60	Tag	–	Piloarrektion
3	24	60	Tag	Ja	Orale Automatismen
4	12	60	Morgen	Ja	Olfaktorische Auren
5	6	45	Morgen	Ja	Aufsteigendes Unwohlsein oder Wärmegefühl und fokale Anfälle mit oralen Automatismen
6	24	60	Morgen	Ja	Areagibilität mit starrem Blick
7	12	30	Morgen	–	Olfaktorische Aura und aufsteigendes Unwohlsein
8	4*	15	Morgen	Ja	Olfaktorische Aura, abdominelle Aura und Piloarrektion
9	24	60	Tag	Ja	Gustatorische Auren und fokale Anfälle mit Areagibilität und oralen Automatismen
10	6*	150	Tag	–	–

*AF* Anfallsfrequenz, *min* Minuten

*Kumulativ

### Klinische und apparative Zusatzdiagnostik

Der Nachweis epilepsietypischer Aktivität im Routine-EEG gelang in unserem Kollektiv erwartungsgemäß nur selten. Bei 4 Patient*innen wurde eine unspezifische Thetaaktivität über der Temporalregion nachgewiesen. In kernspintomografischen Untersuchungen des Neurokraniums stellten sich bei 3 Patient*innen epileptogene Läsionen im Bereich der temporomesialen Strukturen dar, bei 2 Patient*innen links, bei 1 Patienten rechts. In Video-EEG-Langzeitableitungen von mindestens 24 h Dauer wurde bei 6 Patient*innen eine im Schlaf aktivierte epilepsietypische Aktivität über der Temporalregion nachgewiesen, bei 4 Patient*innen über der linken Temporalregion, bei 2 Patient*innen temporal beidseits (Tab. 3). Einmalig wurde ein Anfallsmuster temporal rechts während einer TEA nach dem Erwachen aufgezeichnet (Fall 6). Die ergänzende neuropsychologische Untersuchung erbrachte bei 8 Patient*innen Hinweise auf temporale Funktionsstörungen, welche bereits auch subjektiv von den meisten Patienten wahrgenommen wurden. Bei 3 Patient*innen korrelierte die neuropsychologische Seitenlokalisation mit der Seite des spezifischen Herdbefunds mit Nachweis epilepsietypischer Potenziale (2 links, 1 rechts). Hinweise auf eine depressive Störung fanden sich bei 6 Patient*innen. In einer ergänzenden Liquoranalyse war bei 3 von 10 Patient*innen eine Eiweißerhöhung nachweisbar. Bei 2 von 10 Patient*innen wurden identische Bandenmuster im Serum und Liquor als Hinweis auf eine systemische Immunreaktion nachgewiesen, davon einmalig mit Nachweis einer intrathekalen IgG-Synthese. Die ergänzende Antikörperdiagnostik ergab bei 1 von 10 Patient*innen den schwachen Nachweis von GAD65-Antikörpern. Zudem fielen bei 2 von 10 Patient*innen Bindungsmuster an Neuronen des Hippocampus sowie bei 1 weiteren Patientin Zusatzbanden im Western Blot auf. Eine ergänzende Tumorsuche wurde nur bei 4 von 10 Patient*innen durchgeführt und ergab dort jeweils keinen Hinweis auf eine Tumorerkrankung (Tab. 3).

**Table 3:** 

Fall	cMRT	Ruhe-Wach-EEG	Video-EEG	NPT: temporale Störung	NPT: Depression	Liquoranalyse	Antikörper	Tumorsuche	AED-Effekt (letztes Follow-up)	AED/spez. Therapie
1	Signalanhebung Hippocampus links	Theta temporal beidseits, SW temporal links	SW frontotemporal links (im Schlaf)	Links	+	Eiweißerhöhung	–	–	AF (24 Monate)	LTG, Kortison, IA Azathioprin
2	Signalanhebung Hippocampus und Amygdala links	Theta temporal links	SW temporal anterior links (im Schlaf)	Beidseits (links betont)	+	–	–	–	AF (12 Monate)	LTG
3	Signalanhebung temporomesial rechts	–	Thetaaktivität temporal rechts (MLE)	Links	+	Eiweißerhöhung Ident. Banden S + L	– (Bindungsmuster Hippocampus)	n.e.	AF (24 Monate)	LTG
4	–	–	–	Links	+	n.e.	n.e.	n.e.	? (–)	LTG
5	–	–	SW temporal links (im Schlaf)	–	–	–	–	n.e.	? (–)	LTG
6	–	–	Iktal: Thetarhythmisierung temporal rechts (aus dem Schlaf) Interiktal: SW temporal re>li (im Schlaf)	Rechts	+	–	–	–	AF (6 Monate)	LTG
7	–	Theta temporal links	SW temporal anterior beidseits (rechts nur im Schlaf)	Rechts	+	Intrathekale IgG-Synthese Ident. Banden S + L	– (Zusatzbanden Western Blot)	–	VA (6 Monate)	LEV, LCM, LTG, OXC, PER
8	–	–	–	Links	–	Nicht erfolgt	– (Bindungsmuster Hippocampus)	n.e.	AF (3 Monate)	LEV
9	–	–	–	Links	–	Eiweißerhöhung	GAD65 schwach positiv	n.e.	AF (3 Monate)	LTG
10	–	Theta frontotemporal links	SW temporal links (im Schlaf)	–	–	–	– (Bindungsmuster Hippocampus)	n.e.	? (–)	LTG

*+* leichtgradige Störung, *–* keine Störung, *?* unbekannt, *n.e.* nicht erfolgt, *AED* Antiepileptika, *AF* Anfallsfreiheit, *IA* Immunadsorption, *L* Liquor, *LCM* Lacosamid, *LEV* Levetiracetam, *LTG* Lamotrigin, *MLE* Mobiles Langzeit-EEG, *NPT* neuropsychologische Testung, *OXC* Oxcarbazepin, *PER* Perampanel, *S* Serum, *SW* „sharp waves“, *VA* verbesserte Anfallskontrolle

### Medikamentöse Therapie und Outcome

Bei 9 Patient*innen wurde Lamotrigin zur antiepileptischen Therapie eingesetzt (Tab. 3). Dies verbesserte die Anfallskontrolle bei 5 Patienten. Eine Patientin profitierte nicht von der Lamotrigingabe. Diese Patientin erfüllte die Kriterien einer therapieresistenten Epilepsie (keine Anfallsfreiheit unter Anwendung von mehr als 2 Antiepileptika). Ein weiterer Patient erreichte mit dem Einsatz von Levetiracetam eine gute Anfallskontrolle. Die Anfallskontrolle eines Patienten stabilisierte sich schon zuvor unter Durchführung einer immunsuppressiven Therapie mit Kortison, Immunadsorption und Therapie mit Azathioprin. Die antiepileptische Therapie war danach überlappend von Levetiracetam auf Lamotrigin umgestellt worden, worunter schließlich Anfallsfreiheit erreicht wurde. Bei 3 Patienten erfolgten keine Nachfolgeuntersuchungen mehr an unserer Einrichtung, sodass der Therapieeffekt unbekannt blieb.

## Diskussion

Die transiente epileptische Amnesie ist ein seltenes und wahrscheinlich häufiger übersehenes Phänomen, das vorwiegend bei Patient*innen mit Temporallappenepilepsien auftritt, und seltener auch bei Epilepsien mit extratemporal gelegenen Anfallsgeneratoren [6, 11]. Die Mechanismen, die zur TEA führen, sind nicht abschließend geklärt, aber es gibt Hinweise sowohl auf eine unmittelbare iktale Störung der Gedächtnisleistung als auch Hinweise auf ein postiktales Symptom als Ausdruck einer den Anfall überdauernden passageren Funktionsstörung gedächtnisrelevanter Netzwerke [1]. Zumindest weist das Ansprechen auf Antiepileptika darauf hin, dass epileptische Anfälle ursächlich beteiligt sind. Die Datenlage zur Inzidenz der TEA ist spärlich, mutmaßlich auch wegen der geringen Bekanntheit des Phänomens. Eine prospektive Studie aus einem Zentrum für Demenzerkrankung erbrachte eine Prävalenzrate von 4 % unter den Erkrankten [5]. In unserer retrospektiven Analyse wurden im Jahr 2020 insgesamt 4 von 827 Patient*innen mit einer TEA durch die Schlagwortsuche identifiziert. Wir führten eine ergänzende Stichprobenerhebung durch, um einzuschätzen, wie viele weitere TEA-Fälle durch unsere Suchstrategie (Suchbegriffe „epileptische Amnesie“, „transiente epileptische Amnesie“) fälschlicherweise nicht erfasst wurden. Dabei trafen die TEA-Kriterien bei 2 weiteren Patient*innen von 260 konsekutiven Fällen im Zeitraum von Januar bis einschließlich März 2020 zu, was etwa 0,8 % der erhobenen Stichprobe in einem spezialisierten Epilepsiezentrum entspricht. Zudem waren bei 2 weiteren Patient*innen mit einer Temporallappenepilepsie wenige Minuten anhaltende Episoden mit Desorientierung und Verwirrtheit beschrieben worden, wobei eine sichere Zuordnung zu einer TEA aufgrund fehlender weiterer Angaben nicht möglich war.

### Merkmale der TEA im Kontext vorangegangener Studien

Das Durchschnittsalter unserer Fallserie bei Erstmanifestation liegt bei 59 Jahren und bei Diagnosestellung bei 61 Jahren, ähnlich wie in vorangegangenen Fallserien [1, 2]. Neun von 10 Patient*innen sind männlichen Geschlechts. Eine frühere Fallserie von Zeman aus dem Jahr 1998 kommt zu einem gleich hohen Anteil an männlichen Erkrankten [17]. Weitere Studien zeigen eine Präferenz des männlichen Geschlechts [1, 2]. Der relativ hohe Anteil an Komorbiditäten (vgl. Tab. 2: Gedächtnisstörungen bei 8 von 10 Patient*innen, Depressionen bei 7 von 10 Patient*innen) ist durch die zugrunde liegende chronische Erkrankung des Temporallappens zu erklären. In vorherigen Studien sind Gedächtnisstörungen bei bis zu 80 % der Patient*innen mit TEA sowie Angststörung und depressive Störung bei 20–40 % der Patient*innen mit TEA beschrieben [1, 2, 4, 12].

In unserer Fallserie traten zwischen 4 und 36 Episoden mit TEA pro Jahr auf. Im Gegensatz zur TGA ist die Dauer der TEA kürzer und hält häufig maximal 1 h an (in unserer Fallserie im Durchschnitt 56 min; [1, 2]). Diese Beobachtung ist zum einen als differenzialdiagnostisches Merkmal verwertbar und weist zum anderen darauf hin, dass die TEA sowohl als iktale als auch postiktale Funktionsstörung aufgefasst werden kann. An dieser Stelle sei nochmals hervorgehoben, dass die TEA kein nichtkonvulsiver Status epilepticus ist, wie unter anderem das Fallbeispiel 6 illustriert.

In früheren Studien wurde bereits ein gehäuftes Auftreten am Morgen und nach dem Erwachen als typisches Merkmal beschrieben, was auch in unserer Fallserie, Letzteres bei 6 von 10 Patient*innen, nachvollzogen werden konnte und bei der Diagnosestellung möglicherweise als schwacher Hinweis für eine TEA mehr berücksichtigt werden sollte [1, 2]. Eine Aktivierung des Anfallsgenerators im Schlaf erscheint eine naheliegende Ursache zu sein. In unserem Kollektiv konnte schlafaktivierte epilepsietypische Aktivität bei allen 6 Patient*innen mit Nachweis epilepsietypischer Aktivität beobachtet werden. Dies unterstreicht den zusätzlichen Nutzen einer Langzeit-EEG-Ableitung mit Aufzeichnung eines EEG im Schlaf.

Bei nahezu allen Patient*innen (9 von 10 Patient*innen) ergab eine gezielte epileptologische Anamnese Hinweise auf weitere Anfallstypen. Daher scheint dies neben dem Nachweis epilepsietypischer Aktivität im EEG ein herausragender Aspekt in Hinblick auf die Diagnosestellung zu sein. Sechs von 10 Patient*innen erreichten unter antiepileptischer Therapie eine Anfallsfreiheit. Dies stützt zum einen die Diagnose und spricht für die gute Therapiemöglichkeit dieser Anfallsform, die auch schon in früheren Studien berichtet wurde [1, 2, 17].

Bei 3 von 10 Patient*innen konnte kernspintomografisch eine potenzielle epileptogene Läsion im Bereich der temporomesialen Strukturen nachgewiesen werden, sodass die Durchführung einer Kernspintomografie des Neurokraniums eine sinnvolle Zusatzuntersuchung darstellt und der Abgrenzung konkurrierender Ursachen dient (Rezidiv einer transienten globalen Amnesie oder einer Ischämie). Im Falle einer TGA gelingt kernspintomographisch in bis zu 80 % der Fälle der Nachweis einer flüchtigen punktförmigen Diffusionsstörung des lateralen Hippocampus mit maximaler Ausprägung 48 h nach Symptombeginn [11, 16].

### Differenzialdiagnostische Merkmale der TGA

Die klinische Differenzierung zur TGA oder zu „Verwirrtheitsepisoden“ bei älteren Menschen ist unter anderem deswegen erschwert, weil bei bis zu einem Viertel der TEA-Patient*innen die transiente Amnesie das einzige Symptom der zugrunde liegenden Temporallappenepilepsie sein kann [1]. Eine klinische Abgrenzung zu der in der Bevölkerung häufiger, jedoch selten rezidivierend auftretenden TGA (Rezidivrisiko von circa 5,8 % pro Jahr [14]) ist anhand semiologischer Aspekte bei Erstmanifestation schwierig, was die hohe zeitliche Latenz zwischen Erstmanifestation und Diagnosestellung (vgl. Tab. 2: 22 Monate) und eine vermutlich relativ niedrige Inzidenzrate mit einer wahrscheinlich höheren „Dunkelziffer“ an Fehldiagnosen erklärt [11].

Typische Merkmale der TGA wurden von Caplan 1985 sowie Hodges und Warlow 1990 beschrieben ([3, 7]; s. Infobox 2).

#### Infobox 2 Diagnosekriterien der TGA nach Caplan (1985) und Hodges und Warlow (1990)

- Akute und ausgeprägte Neugedächtnisstörung über mindestens 1 h mit Rückbildung innerhalb von 24 h
- Kein Vorliegen fokal neurologischer Defizite, zusätzlicher kognitiver Defizite, Bewusstseinstrübungen oder Desorientierung zur Person
- Kein vorrausgehendes Trauma oder bekannte Epilepsie

Begleitend können Übelkeit, Schwindel und Kopfschmerzen in leichter bis mäßiger Ausprägung auftreten. Zusatzuntersuchungen wie eine cMRT- und EEG-Untersuchung (unspezifische Theta- oder Deltawellen temporal [8]) können im Einzelfall zur unterstützenden Differenzialdiagnostik sinnvoll sein. Komorbiditäten wie Depressionen oder chronische Gedächtnisstörungen sind bei der TGA nicht beschrieben. Betroffene sind häufig über 50 Jahre alt. In der Literatur ist ein gehäuftes Auftreten nach einer emotionalen Belastung, einem Sprung ins kalte Wasser, körperlicher Belastung oder Geschlechtsverkehr sowie eine tageszeitliche Häufung am Vormittag beschrieben [15, 16]. Die Akutphase überdauernde, lang anhaltende Gedächtnisdefizite wurden bei der TGA nicht beobachtet. Angststörungen und depressive Störungen finden sich bei 20–25 % der Patient*innen mit TGA [9, 13]. Eine Gegenüberstellung der klinischen Merkmale zwischen TGA und TEA ist in Tab. 4 zusammengefasst.

**Table 4:** 

Merkmal	TEA	TGA
Inzidenz	Unbekannt	3–8/100.000 Einwohner/Jahr
Alter bei Erstmanifestation	> 50 Jahre	> 50 Jahre
Dauer	15–60 min	1–24 h
Rezidive	1/Monat	5,8 %/Jahr
Häufige Begleitumstände bzw. Auslöser	Erwachen aus dem Schlaf	Emotionale Belastung Körperliche Anstrengung Sprung ins kalte Wasser Geschlechtsverkehr
Typische Begleitsymptomatik	Olfaktorische Auren Orale/manuelle Automatismen Weitere temporale Elemente	Übelkeit Schwindel Kopfschmerz
Tageszeitlicher Schwerpunkt	Morgens	Vormittags
MRT	Selten epileptogene Läsion	Punktförmige DWI/T2-Läsion lateraler Hippocampus mit maximaler Ausprägung 48–72 h nach Symptombeginn
EEG	Epilepsietypische Potenziale über dem betroffenen Temporallappen (bei ca. 1/3 der Patienten)	Unspezifische Theta‑/Deltaaktivität temporal
Ansprechen auf AED	Ja	Nein
Persistierende Gedächtnisstörung	Akzeleriertes Vergessen Autobiographisches Gedächtnis Topographisches Gedächtnis	–
Angststörung/depressive Störung	20–40 %	20–25 %

*DWI* Diffusionsgewichtete Sequenzen in kernspintomographischen Aufnahmen, *TEA* transiente epileptische Amnesie, *T2, T2* gewichtete Sequenzen

### Diagnosestellung der TEA

Die Diagnose einer TEA kann in Analogie zur Diagnose einer Epilepsie klinisch anhand typischer anamnestischer Hinweise beim wiederholten Auftreten (vgl. Tab. 4) oder beim Nachweis epilepsietypischer Aktivität im EEG bzw. einer typischen epileptogenen Läsion in der Kernspintomografie des Neurokraniums erfolgen. Bei Patient*innen ohne zusätzliche Hinweise auf eine Epilepsie kann das Ausbleiben der rezidivierenden Episoden mit Amnesie auf Gabe von Antiepileptika die TEA-Diagnose unterstützen. Die Durchführung einer Langzeit-EEG-Untersuchung erhöht die Wahrscheinlichkeit, epilepsietypische Aktivität vor allem im Schlaf zu erfassen.

Weitere diagnostische Schritte wie Untersuchungen des Liquors, anti- und onkoneuronaler Antikörper und eine erweiterte Tumorsuche dienen einer ätiologischen Zuordnung der Temporallappenepilepsie und erscheinen für die Diagnosestellung im Einzelfall sinnvoll, nicht jedoch in der klinischen Routine. Die Durchführung einer neuropsychologischen Untersuchung mit Frage nach temporalen Funktionsstörungen ist für die Diagnosestellung nicht zwingend erforderlich, vermag jedoch die bereits häufig subjektiv berichteten Gedächtnisstörungen zu objektivieren. Eine Studie von Lanzone aus dem Jahr 2020 zeigte, dass es bei Patient*innen mit TEA Veränderungen der Frequenzbänder in der EEG-Spektralanalyse mit Zunahme im Betaband gibt. Möglicherweise ist dies als Ausdruck einer chronischen Veränderung des Temporallappens bei Patient*innen mit TEA zu werten und könnte bei der Differenzierung zwischen TEA und TGA hilfreich sein [10].

Spätestens beim zweiten Auftreten einer passageren Episode mit amnestischer Störung sollte differenzialdiagnostisch an eine TEA gedacht werden und ggf. eine weitere epileptologische Diagnostik veranlasst werden, um das Rezidivrisiko und das Auftreten von weiteren, häufig unbewusst erlebten fokalen Anfällen sowie das Auftreten von anfallsassoziierten Verletzungen und Unfällen zu reduzieren. Vor diesem Hintergrund scheint eine frühzeitige Diagnosestellung von hoher Bedeutung für den weiteren Verlauf der Erkrankung und das Wohlergehen der Erkrankten zu sein.

## Fazit für die Praxis

- Eine transiente epileptische Amnesie (TEA) kann klinisch anhand typischer klinischer Merkmale (wiederholtes Auftreten, zusätzliche anfallsassoziierte Symptome, Dauer ≤ 60 min) diagnostiziert werden.
- Nach Hinweisen auf ursächliche Temporallappenepilepsie sollte gefahndet werden.
- EEG- und cMRT-Untersuchungen sind diagnostisch hilfreich.
- Ein Ansprechen auf Antiepileptika unterstützt die Diagnose.
- Im Einzelfall sind Liquor- und Antikörperuntersuchungen, eine neuropsychologische Untersuchung sowie nuklearmedizinische Untersuchungen sinnvoll.
